# Investigating the role of SARM1 in central nervous system

**DOI:** 10.1002/ibra.12173

**Published:** 2024-08-14

**Authors:** Junjie Wang, Yuhang Shi, Jinglei Tian, Liming Tang, Fang Cao

**Affiliations:** ^1^ Department of Neurosurgery Affiliated Hospital of Zunyi Medical University Zunyi China

**Keywords:** brain, central nervous system disease, SARM1, Wallerian degeneration

## Abstract

Sterile‐α and Toll/interleukin 1 receptor (TIR) motif‐containing protein 1 (SARM1), a key intracellular molecule that plays numerous important biological functions in the nervous system, has attracted much attention. Recent studies have shown that SARM1 plays a key role in nerve injury, degeneration, and neurodegenerative diseases. Therefore, understanding the role of SARM1 in the central nervous system (CNS) will enhance our knowledge of the pathogenesis of CNS diseases and aid in the development of new therapeutic strategies. This review will explore the biological functions of SARM1 in the nervous system and its potential roles in nerve injury and disease, thus providing new directions for future research and treatment.

## Graphical abstract

Sterile‐α and Toll/interleukin 1 receptor (TIR) motif‐containing protein 1 (SARM1) is a pivotal molecule that has garnered extensive attention in neuroscience. As an intracellular molecule, SARM1 possesses various crucial biological functions in the nervous system. Recent studies have demonstrated that SARM1 plays a pivotal role in nerve injury, degenerative diseases, and neurodegenerative diseases. Exploring the role of SARM1 in the central nervous system (CNS) allows us to gain a deeper understanding of the pathogenesis of CNS diseases and explore novel therapeutic strategies. In this study, we will investigate the biological functions of SARM1 in the nervous system and its potential implications in nerve injury and related diseases, thereby offering novel perspectives for future research and treatment.

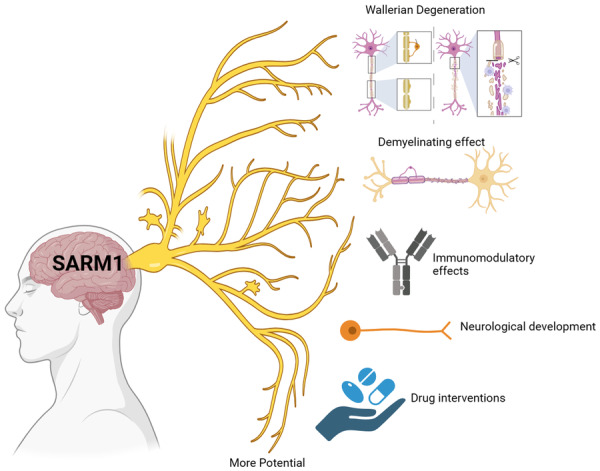

## 1 INTRODUCTION

With the advancement of scientific knowledge, genetic medicine is widely utilized to diagnose, treat, and prevent diseases. It encompasses various approaches, such as gene replacement, gene repair, gene knockout, and gene editing. These techniques hold promise in treating a wide range of hereditary and infectious diseases, including hemophilia, cystic fibrosis, and acquired immunodeficiency syndrome (AIDS), among others.^1^, ^2^, ^3^ For example, gene editing using CRISPR/Cas9 has shown great potential.^4^ It is also reported that a specific genetic medicine approach employing a retrovirus (RV) as a vector to deliver functional genes into a patient's blood stem cells can target adenosine deaminase deficiency (ADA), a genetic disorder.^5^ Within the central nervous system (CNS), scientists have been extensively studying the gene sterile‐α and Toll/interleukin 1 receptor (TIR) motif‐containing protein 1 (SARM1) because it has emerged as a key player in diverse CNS disorders. Therefore, this review focuses on elucidating the role of SARM1 in the CNS and presenting the latest advancements in research.

## 2 OVERVIEW OF SARM1

**Figure 1:**
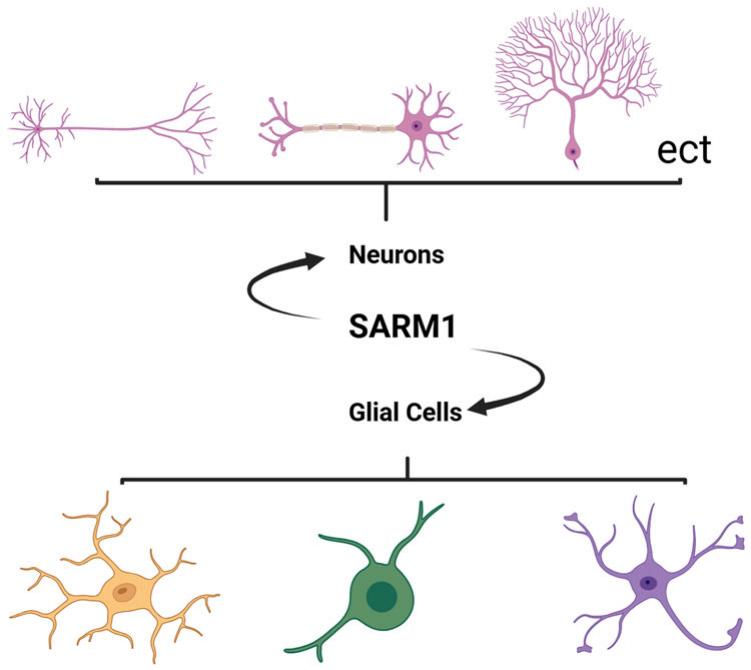
Distribution of sterile‐α and Toll/interleukin 1 receptor (TIR) motif‐containing protein 1 (SARM1) in the brain. SARM1 is predominantly distributed in neurons (including cortical neurons in the cerebral cortex, pyramidal neurons, and granule cells in the hippocampus; neurons in the caudate nucleus and putamen of the striatum; motor neurons and sensory neurons in the spinal cord; neurons in various nuclei of the brainstem; Purkinje cells; and others in the cerebellum) and glial cells (including astrocytes, oligodendrocytes, microglia) (created with BioRender.com). [Color figure can be viewed at wileyonlinelibrary.com]

SARM1 is a protein that interacts with Toll‐like receptors at the junction.^6^ It is the most conserved member of the Toll/interleukin 1 receptor (TIR) junction protein family and is highly expressed in the peripheral nervous system (PNS) and CNS. It is reported that SARM1 is widely expressed in numerous tissues, predominantly at elevated levels within the brain (Figure 1), and to a lesser degree in the kidney and liver. Its expression in the placenta is relatively lower. Experimental evidence from Fazal et al. demonstrates that it is primarily expressed in glial cells (particularly oligodendrocytes), immune cells, and neurons within the nervous system.^7^

SARM1 plays a critical role in Wallerian degeneration and is involved in neuroinflammation and the development of the nervous system. More importantly, it is responsible for mediating neuronal death and driving morphological changes. It also regulates Wallerian degeneration in nerve fibers and influences gliogenesis. Deletion of SARM1 has been shown to provide significant protection against axonal degeneration or loss in conditions such as traumatic brain injury (TBI) and chemometabolic neuropathy.^8^, ^9^, ^10^, ^11^ Thus, the prospect of knocking down or inhibiting SARM1 through techniques like gene biotechnology and pharmacological interventions is highly appealing for prognostic investigations of different neurological disorders (Table 1).

**Table 1:** Effects of sterile‐α and Toll/interleukin 1 receptor (TIR) motif‐containing protein 1 (SARM1) in the central nervous system.

Disease/intervention	Effect	References
Axonal damage	SARM1 may cause neuronal damage by mediating the process of axonal death.	[12, 13, 14]
Alzheimer's disease	SARM1 may be involved in the neuronal death process and may be associated with neuronal damage in Alzheimer's disease.	[15, 16, 17]
Parkinson's disease	SARM1 may be involved in the pathogenesis of Parkinson's disease by regulating immune responses and inflammatory processes and oxidative stress.	[16, 17, 18]
Multiple sclerosis	SARM1 may be involved in neuronal axonal damage and demyelination in MS (multiple sclerosis).	[19, 20, 21]
Drug interventions	SARM1 inhibition with small molecules has the potential to treat axonopathies of the central and peripheral nervous systems by preventing axonal degeneration and by allowing functional recovery of a metastable pool of damaged, but viable, axons.	[12, 22, 23]

## 3 MOLECULAR STRUCTURE OF SARM1

**Figure 2:**
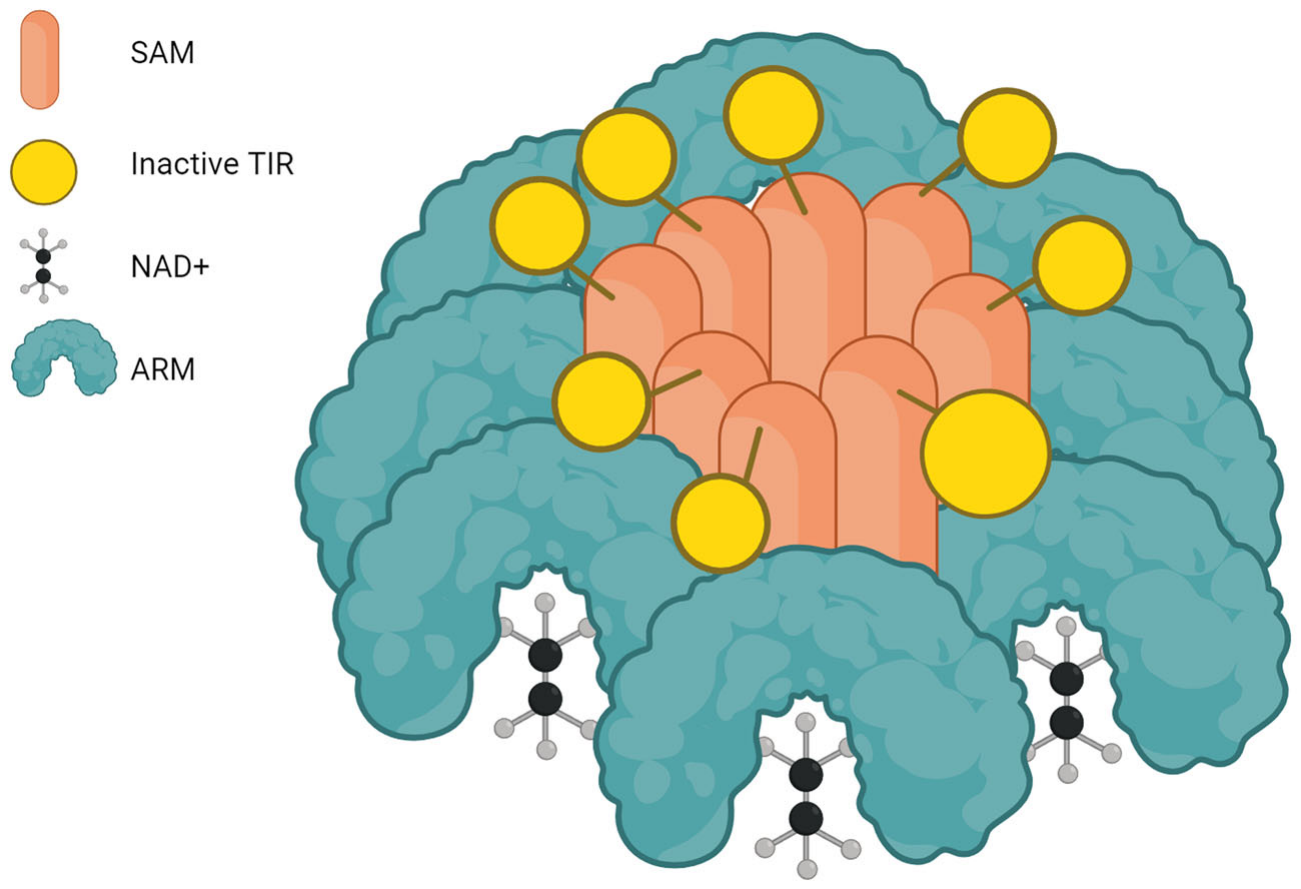
Molecular structure of sterile‐α and Toll/interleukin 1 receptor (TIR) motif‐containing protein 1 (SARM1). Sterile alpha motif (SAM) is situated at the core of SARM1, whereas the TIR domain resides at the C‐terminus, nicotinamide adenine dinucleotide (NAD^+^) acts as a substrate for SARM1, and the armadillo repeat (ARM) domain is positioned at the N‐terminus. Within the octameric structure of SARM1, the ARM domain plays a crucial supportive role by promoting the formation and ensuring the stability of the octamers. The conjunction of SAM and the TIR domain is involved in the formation and maintenance of octamers (created with BioRender.com). [Color figure can be viewed at wileyonlinelibrary.com]

SARM1 is a complex protein with multiple structural domains that is primarily located in the outer membrane of mitochondria. It consists of different domains, including an auto‐inhibitory armadillo repeat (ARM) domain, a tandemly oligomerized sterile alpha motif (SAM) domain, and a catalytic TIR domain.^24^, ^25^ Full‐length SARM1 exists in an inactive state as a preassembled oligomer consisting of eight subunits, which exhibit autoinhibitory properties^26^ (Figure 2). The key “ARM–TIR” lock is the molecular regulator of the inactive state, which acts as the interface between the autoinhibitory N‐terminal ARM domain and the catalytic C‐terminal TIR domain. In the inactive conformation, this structural domain functions as a catalyst for TIR trapping. The lock prevents the TIR domains from approaching each other closely, thereby preventing the formation of an effective active site. Altering specific key amino acids in the ARM structural domains can open this lock, enabling the already assembled complex to become catalytically active and induce severe axonal damage. The multimerization of TIR's structural domains seems to be essential for its catalytic activity. The SARM1 protein forms a cyclic octamer, with the ARM structural domain interacting with the TIR structural domain to inhibit the hydrolysis of nicotinamide adenine dinucleotide (NAD^+^) by TIR. Through this approach, the SARM1 protein can sustain its own inhibited activity in healthy neurons.^27^

Through an analysis of the NAD^+^ degrading activity kinetics of the full‐length SARM1 protein, Zhang et al. discovered that a high concentration of NAD^+^ (approximately 500 µM) interacts with and stabilizes the structure of ARM in healthy neuronal cells.^27^ This interaction enables ARM to interact with TIR, resulting in the inhibition of NAD^+^ hydrolyzing activity of TIR. Physical injury or pathological stimuli can partially decrease NAD^+^ levels in axons and dissociate NAD^+^ bound to ARM. Consequently, this weakens the stability of the domain structure and its binding ability to TIR, resulting in the release of TIR's NAD^+^ hydrolyzing activity and ultimately leading to the rapid degradation of NAD^+^.

## 4 MAIN ROLES OF SARM1 ON THE CNS

### 4.1 Neuronal damage and axonal severance

The transmission of information between neurons is dependent on the length of their axons, which can vary based on factors such as species, neuron type, and target cell. Put differently, axons can account for more than 99.9% of a neuron's total volume.^28^ From a structural perspective, the axon consists of short actin filaments.^29^ One end of these filaments is coated with endoreduplication and arranged in a circular pattern, encircling the entire circumference of the axon.^30^, ^31^ Axonal degeneration leads to the loss of neurological function, setting it apart from classical forms of cell death such as apoptosis, pyroptosis, and necroptosis.

**Figure 3:**
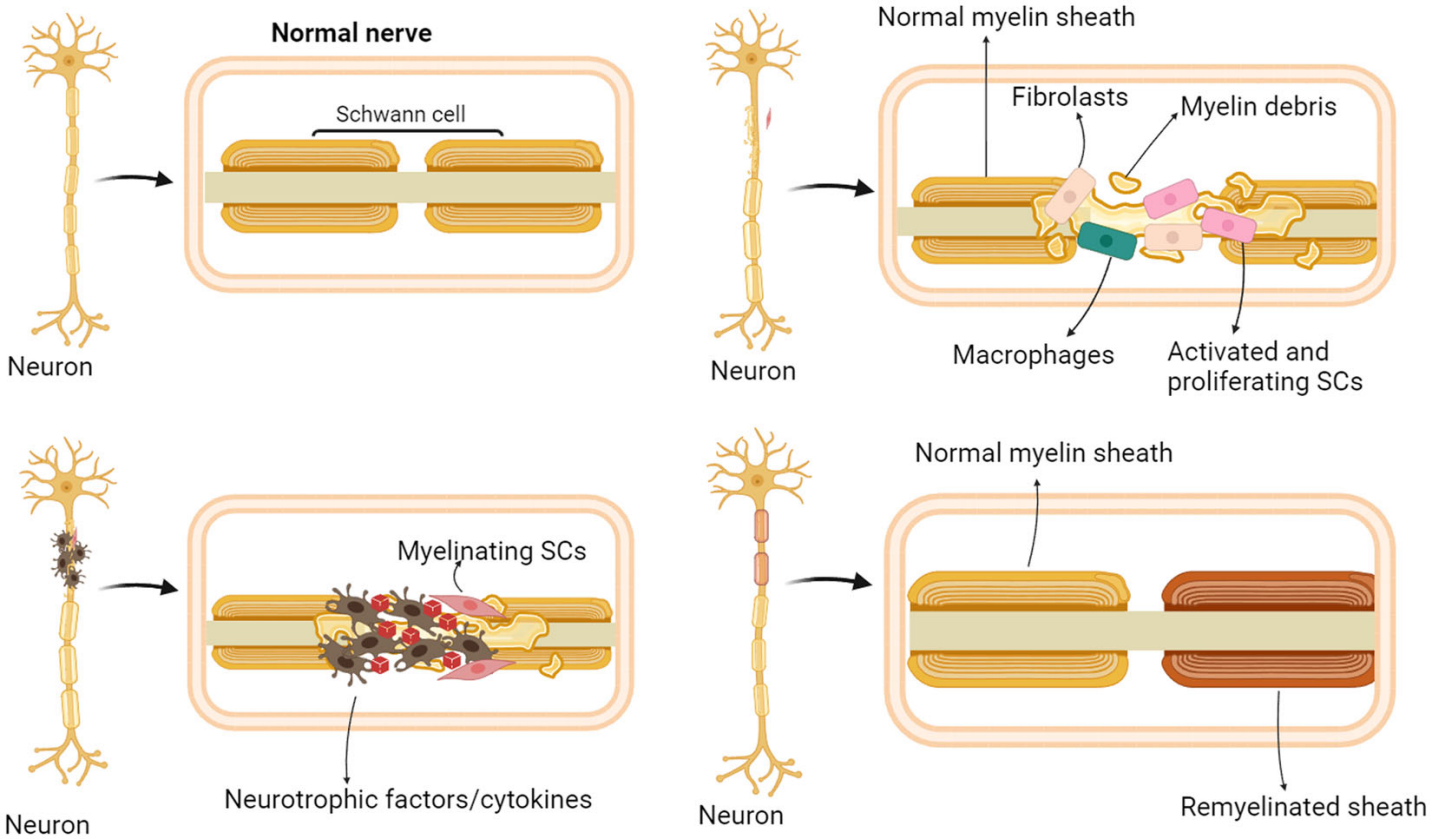
Wallerian degeneration. The surrounding glial cells and neighboring cells react rapidly when an axon is damaged. This encompasses the migration of macrophages and other immune cells to the damaged area, as well as the proliferation and activation of neighboring neuroglia. The formation of the Wallerian degeneration zone, characterized by inflammation and degeneration, marks the onset and reaction to the injury. As a result of the injury, the myelin sheath surrounding the axon begins to rapidly break down and disintegrate. The disintegration of the myelin sheath exposes the axon to the external environment, increasing its sensitivity to external stimuli. With the progression of the injury, the axoplasm (containing mitochondria, organelles, and cytoplasm) within the axon undergoes degeneration and eventually collapses. Simultaneously, glial cells and immune cells in the surrounding area may remove residual axonal debris, thereby facilitating the onset of axonal regeneration (created with BioRender.com). [Color figure can be viewed at wileyonlinelibrary.com]

**Figure 4:**
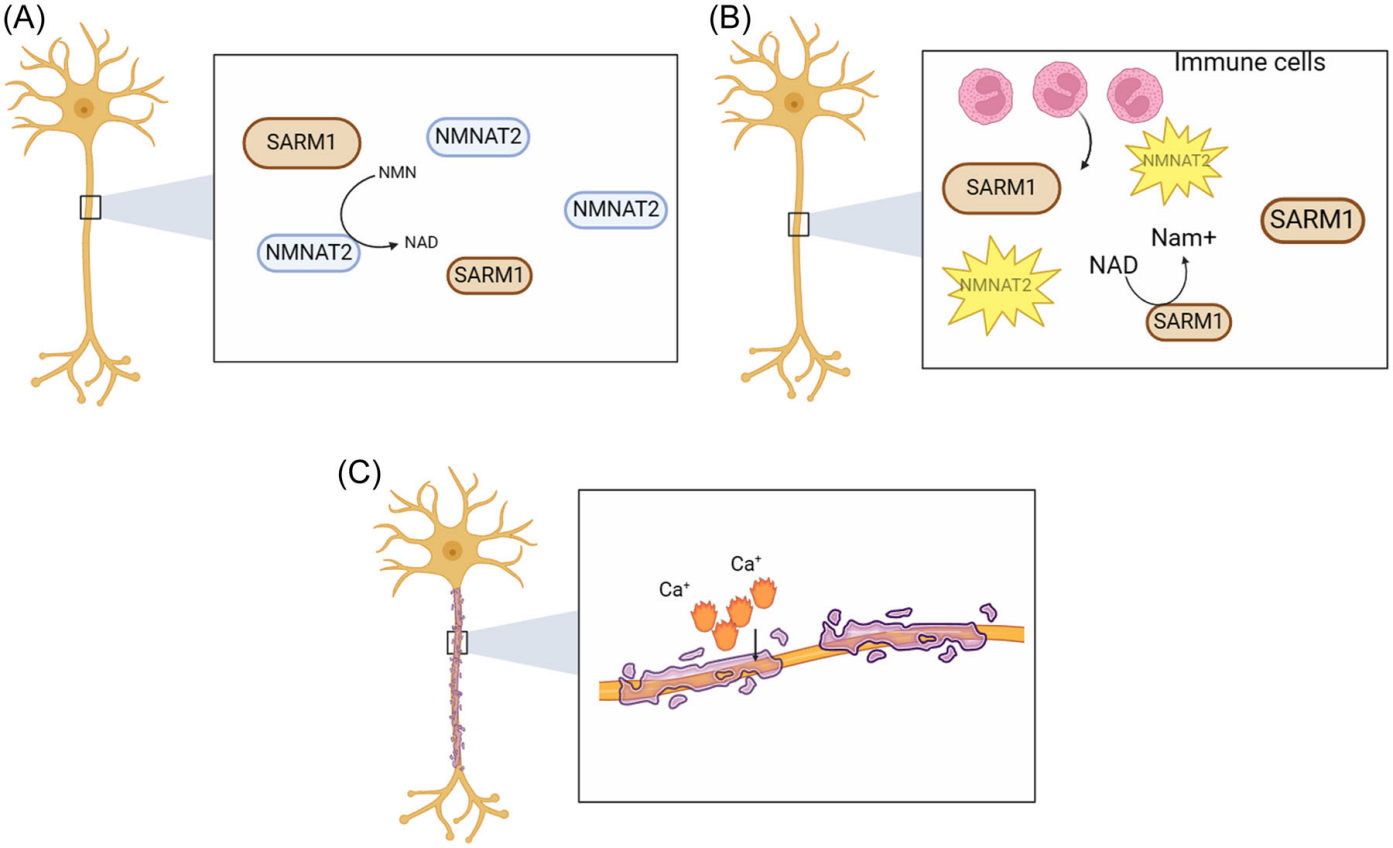
The activation pathways of sterile‐α and Toll/interleukin 1 receptor (TIR) motif‐containing protein 1 (SARM1). (A) Survival‐promoting factor nicotinamide mononucleotide adenylyltransferase 2 (NMNAT2) is transported along the axon to maintain high levels of nicotinamide adenine dinucleotide (NADH) and low levels of nicotinamide mononucleotide (NMN) promote degenerative SARM1 with only basic activity. (B) Injury results in the suspension of NMNAT2 transport along axons, NMNAT2 is unstable and breaks down quickly with decreased NAD, and elevated NMN SARM1 is activated by nonmicrobial NAN (NMNAN) and degrades NAD. The axon AT2 appears intact but will degrade. (C) Ca^2+^ intracavity causes SARM1 activation (created with BioRender.com). [Color figure can be viewed at wileyonlinelibrary.com]

Over a century ago, Augustus Waller discovered that the glossopharyngeal and hypoglossal nerves in frogs undergo degenerative changes following injury. He also observed that the myelin sheaths broke down 12–15 days after the nerves were severed. In subsequent studies, Waller discovered that the nerve regenerated within the tongue after 3–4 months, demonstrating the sustained activity of the nerve cells. The breakdown of the myelin sheath does not follow a proximal‐to‐distal pattern. Instead, it initiates at the junction of Schwann's cells and further undergoes degenerative resorption around the nodes of Ranvier, leading to the complete elimination of the myelin sheath.^32^, ^33^ Therefore, Wallerian degeneration refers to a sequence of metaplastic and cytotoxic processes involving axonal necrosis, myelin disintegration, and proliferation of the nerve sheath membrane. It occurs in the distal segment of neurons following neuronal injury, starting with rapid axonal and myelin disintegration. This is then followed by microglia activation, clearance of tissue debris, and glial cells' proliferation^34^ (Figure 3). Two critical regulatory influences that play a significant role in Wallerian degeneration are the upstream pro‐survival regulators: nicotinamide mononucleotide adenylyltransferase 2 (NMNAT2) and SARM1. Neuronal axon damage leads to cell membrane integrity impairment, disrupting the intracellular and extracellular ionic balance and elevating intracellular calcium ions. It is a common indicator of neuronal stress and injury.^35^ High intracellular calcium ion concentrations can activate SARM1 activity^36^ (Figure 4).

SARM1 is primarily located in the outer mitochondrial membrane. Upon activation, SARM1 acquires NADase activity, which is only observed in response to brain injury or trauma.^37^ It cleaves NAD, an essential molecule for mitochondrial function. NAD^+^ serves as a critical intracellular coenzyme, participating in various biological processes, particularly energy metabolism. Intracellularly, NAD^+^ participates in various essential reactions such as glycolysis, the mitochondrial respiratory chain, DNA repair, and poly ADP‐ribose polymerase (PARP)‐mediated DNA repair.^38^ Decreased levels of NAD^+^ in the body, tissues, cells, and mitochondria result in increased production of reactive oxygen species (ROS) and a reduction in oxidative metabolism. This leads to an elevated NADH/NAD^+^ ratio and impacts mitochondrial function. Sasaki et al. discovered that SARM1 activation occurs in response to axon damage, leading to NAD depletion and eventual axon breakage.^37^ Nicotinamide mononucleotide adenylyltransferase (NMNATase) and nicotinamide mononucleotide (NMN) deamidase prevent SARM1‐induced NAD depletion, and they offer robust axonal protection similar to SARM1‐deficient axons. Among these, NMN deamidase is an enzyme that depletes NMN. Nicotinamide phosphoribosyltransferase (NAMPT) or nicotinamide riboside kinase (NRK) + nicotinamide riboside (NR) can increase intracellular NAD levels in axons before injury, thereby delaying the NAD levels from reaching critical thresholds and preventing subsequent axonal degeneration.^39^ This is similar to the relationship between toxins and antitoxins. In this case, NMNAT2 inhibits the prodegenerative activity of SARM1, just as antitoxins inhibit the function of toxins. Second, SARM1 and NMNAT2 are genetically related in a classical manner. The deletion of SARM1 does not exhibit any observable phenotype in mice until it is stimulated by an appropriate event, such as neuronal injury.^40^, ^41^

Therefore, when SARM1 is activated, the degradation of NAD^+^ within the nerve cell results in energy depletion. As the axon plays a crucial role in communicating with the target cell, when energy is depleted in the axon region, it results in the disintegration and detachment of the axon structure. Moreover, the decrease in NAD and adenosine triphosphate (ATP) levels causes mitochondrial depolarization and subsequent axonal degeneration.^6^, ^42^ Ultimately, this process results in the degeneration and breakage of the axon.

### 4.2 Demyelinating effect

Demyelination is a condition characterized by the disruption or loss of the myelin sheath that surrounds a nerve fiber. Myelin is a lipid layer that is synthesized by glial cells. It covers the nerve fibers and plays a role in accelerating electrical signaling while also providing protection to the nerve fibers.^43^ Impairment in the function of the nervous system occurs when the myelin sheath is damaged or destroyed, resulting in a slowdown of nerve impulse transmission.

In 2019, Marion et al. reported that in mouse experiments, TBI in SARM1^+/+^ mice led to significant axonal demyelination and folding, whereas SARM1^−/−^ mice did not exhibit notable alterations in myelin formation patterns.^44^ Jin et al. discovered upregulation of SARM1 in astrocytes, which in turn inhibits the expression of glial cell line‐derived neurotrophic factor (GDNF), a neurotrophic factor, through modeling of experimental autoimmune encephalomyelitis (EAE) in mice. Activation of nuclear factor‐kappaB (NF‐κB) signaling by GDNF is believed to contribute to the promotion of neuroinflammation and demyelination.^19^

### 4.3 Immunomodulatory effects

Recent studies have raised questions about the role of SARM1 in the immune response, despite it not being traditionally classified as an immunomodulatory protein. SARM1 is the founding member of the TIR structural domain family of innate immune NADases. It is the only protein within this family that exhibits this activity. Aaron DiAntonio demonstrated that TIR‐mediated NAD cutting is a primitive innate immune function by studying the function of TIR NADases in bacterial, plant, and animal responses to infection.^41^ Recent research indicates that SARM1 plays a role in regulating neuronal intrinsic immune responses and white matter and prion‐induced neuroinflammation.^44^, ^45^ SARM1 activity can influence the immune response and inflammatory processes initiated by an injury. Neurons have been shown to autonomously detect axonal damage in distal regions of the cell, which leads to the rapid production of a specific set of cytokines and chemokines, while the regulation of this immune response is mediated by the TIR structural domain connector SARM1/MyD88‐5. Activation of SARM1 triggers the downstream JNK‐c‐Jun signaling pathway, which, in turn, initiates a cascade of biological responses. These responses involve the activation and recruitment of immune cells in damaged neural tissue. However, inhibition of the SARM1–JNK–c‐Jun pathway effectively prevents the migration of immune cells to damaged neural tissues.^41^ Liu et al. investigated the potential involvement of SARM1 in neuroinflammation after spinal cord injury (SCI). They discovered that when SARM1 was knocked down in neurons and astrocytes, it inhibited neuroinflammation post‐SCI by upregulating HSP70. This upregulation of HSP70 further suppressed the activation of the NF‐κB signaling pathway, ultimately promoting neuronal regeneration following SCI.^46^

### 4.4 Neurological development and degeneration

SARM1 regulates neuronal morphogenesis through at least two pathways. SARM1 controls neuronal morphogenesis through the ASK1–MKK–JNK pathway, including dendritic arborization, axon growth, and the establishment of neuronal polarity.^47^ The expression of SARM1 in neurons also regulates the production of inflammatory cytokines in the brain. This regulation has been observed to have an impact on both brain development and function.^48^

Chen et al. demonstrated that knocking down the expression of SARM1 in hippocampal neurons led to a significant decrease in both dendrite length and dendrite number in the dendritic arborization. This reduction subsequently resulted in a significant decrease in the number of dendritic crossings.^49^ Knocking down SARM1 also had an impact on both the quantity and structure of dendritic spines in mature neurons. In neurons with reduced SARM1 expression, there were still mushroom‐like or stubby dendritic spines, but their density along the dendrites was decreased. Furthermore, the remaining dendritic spines in these neurons were longer and had larger heads compared with neurons transfected with the vector control.^47^

Neurodegenerative diseases involve neuronal damage and partial disassembly of their axons. SARM1 is considered a key regulator in this process. As mentioned earlier, the NADase activity of SARM1 reduces the levels of NAD^+^, resulting in a decrease in redox buffer protection. Interestingly, studies have shown that administering NR to mice with an AD model can increase NAD levels. This approach aims to correct the β‐amyloid levels that are affected by the decline in NAD levels. This intervention leads to an improvement in cognitive function and hippocampal synaptic plasticity in the mouse model of Alzheimer's disease (AD).^50^, ^51^, ^52^ Similarly, in experiments conducted on Parkinson's disease (PD), elevating NAD levels through the administration of niacinamide (NAM) and inhibiting poly‐adenosine diphosphate‐ribose polymerases (PARPs) showed improvements in mitochondrial function and provided protection against neuronal degeneration.^53^

### 4.5 Relevant intervention studies targeting SARM1 and its therapeutic potential

Due to the crucial role of SARM1 in neuropathy, research and drug development efforts have been directed toward the creation of SARM1 inhibitors or intervening agents that target other alterations caused by SARM1. The aim is to treat neurological disorders by slowing down or preventing axon self‐catabolism. As an example, in 2021, Li et al. used immunofluorescence and cryo‐electron microscopy to identify dehydrogenated nitrosoisohexanediol (dHNN) as an inhibitor of SARM1 activation.^53^ dHNN inhibits SARM1 activation through covalent modification of Cys311 and immobilization in an octameric lotus‐like structure, which reveals potent allo‐inhibitory sites. Notably, dHNN is derived from nisoldipine. The metabolic conversion of nisoldipine to dHNN, which leads to SARM1 inhibition, could potentially explain the neuroprotective effects of nisoldipine.^54^ Syntaphilin is another example, a mitochondrial docking protein that functions as a “static anchor” and whose inhibition enhances mitochondrial transport. Thus, Syntaphilin, as a crucial mediator of mitochondrial transport, has the potential to improve axonal regeneration after SCI.^55^ This niacinamide NADase activity, which we previously mentioned as a significant component of the conserved axon death program.

In Drosophila, Sarkar and colleagues validated the restoration of mitochondrial complex I activity in cells treated with the potent mitochondrial complex I inhibitor rotenone through the use of a PARP inhibitor, PJ34. They also demonstrated that this restoration prevented SARM1 activation in the same cells. It is important to note that the SARM1 protein levels were significantly reduced, and the hyperactivation of PARP1 induced by fisetinone occurred before the levels of SARM1‐induced production. This study not only reveals the extent of endogenous NAD^+^ regulation on SARM1 neoinduction but also suggests that PARP inhibitors could offer a new therapeutic approach.^56^

Oxidative stress plays a crucial role in the development and advancement of various degenerative neurological disorders. Cellular stress responses and endogenous antioxidant defense systems are critical for cellular resistance to oxidative damage. SARM1 acquires NADase activity, which regulates mitochondrial health by breaking down NAD^+^. It is believed that SARM1 plays a protective role in degenerative diseases and may impact mitochondrial bioenergetics. SARM1 knockout mice compared with wild‐type mice suggest that SARM1 could hinder the progression of neurodegeneration by controlling mitochondrial respiration, leading to a decrease in oxidative stress mediated by type 2 nuclear respiratory factor (NRF2) and its associated vitamins.^57^, ^58^, ^59^

In combination, the vitamin network induced by phenols may have a significant role in defending against degenerative diseases induced by oxidative stress. The regulation of cellular stress response and redox homeostasis suggests that the vitamin network could serve as a significant target for future therapeutic approaches aimed at neurodegenerative diseases. Future research should investigate these mechanisms more thoroughly and devise therapeutic approaches that specifically target the vitamin network to enhance the antioxidant capacity of the nervous system.

## 5 DEVELOPMENT AND PROSPECTS

In summary, studies on the role of SARM1 in the CNS have yielded significant insights into the mechanisms underlying nerve injury and have opened up new avenues for the development of targeted therapeutic interventions. Researchers are investigating methods to inhibit the activation of SARM1 as a means to safeguard the nervous system against injury. Numerous experimental drugs and therapeutic approaches have demonstrated neuroprotective effects in animal models, bringing hope for the future treatment of neurodegenerative diseases. Nevertheless, there is still ongoing research to fully understand the precise role of SARM1 in human diseases, and further exploration is needed to clinically validate relevant therapeutic strategies.

## AUTHOR CONTRIBUTIONS

Junjie Wang and Yuhang Shi contributed to the conception and design. Junjie Wang, Jinglei Tian, and Liming Tang are responsible for picture production and manuscript drafts. Fang Cao contributed to supervision, proof checking, revision, and editing. All authors have read and approved the final manuscript.

## CONFLICT OF INTEREST STATEMENT

The authors declare no conflict of interest.

## ETHICS STATEMENT

Not applicable.

## Data Availability

Not applicable as no new data are generated in this study.

## References

[1] Dunbar AJ , Rampal RK , Levine R . Leukemia secondary to myeloproliferative neoplasms. Blood. 2020;136(1):61‐70. 10.1182/blood.2019000943 32430500 PMC7332899

[2] Liu C , Zhao W , Zhang L , Sun H , Chen X , Deng N . Preparation of DSPE‐PEG‐cRGD modified cationic liposomes for delivery of OC‐2 shRNA and the antitumor effects on breast cancer. Pharmaceutics. 2022;14(10):2157. 10.3390/pharmaceutics14102157 36297592 PMC9612323

[3] Xu L , Yang H , Gao Y , et al. CRISPR/Cas9‐mediated CCR5 ablation in human hematopoietic Stem/progenitor cells confers HIV‐1 resistance in vivo. Mol Ther. 2017;25(8):1782‐1789. 10.1016/j.ymthe.2017.04.027 28527722 PMC5542791

[4] Wang SW , Gao C , Zheng YM , et al. Current applications and future perspective of CRISPR/Cas9 gene editing in cancer. Mol Cancer. 2022;21(1):57. 10.1186/s12943-022-01518-8 35189910 PMC8862238

[5] Bradford KL , Moretti FA , Carbonaro‐Sarracino DA , Gaspar HB , Kohn DB . Adenosine deaminase (ADA)‐deficient severe combined immune deficiency (SCID): molecular pathogenesis and clinical manifestations. J Clin Immunol. 2017;37(7):626‐637. 10.1007/s10875-017-0433-3 28842866

[6] Essuman K , Summers DW , Sasaki Y , Mao X , DiAntonio A , Milbrandt J . The SARM1 Toll/Interleukin‐1 receptor domain possesses intrinsic NAD(+) cleavage activity that promotes pathological axonal degeneration. Neuron. 2017;93(6):1334‐1343.e5. 10.1016/j.neuron.2017.02.022 28334607 PMC6284238

[7] Fazal SV , Mutschler C , Chen CZ , et al. SARM1 detection in myelinating glia: sarm1/Sarm1 is dispensable for PNS and CNS myelination in zebrafish and mice. Front Cell Neurosci. 2023;17:1158388. 10.3389/fncel.2023.1158388 37091921 PMC10113485

[8] Geisler S , Doan RA , Strickland A , Huang X , Milbrandt J , DiAntonio A . Prevention of vincristine‐induced peripheral neuropathy by genetic deletion of SARM1 in mice. Brain. 2016;139(Pt 12):3092‐3108. 10.1093/brain/aww251 27797810 PMC5840884

[9] Bosanac T , Hughes RO , Engber T , et al. Pharmacological SARM1 inhibition protects axon structure and function in paclitaxel‐induced peripheral neuropathy. Brain. 2021;144(10):3226‐3238. 10.1093/brain/awab184 33964142 PMC8634121

[10] Cheng Y , Liu J , Luan Y , et al. Sarm1 gene deficiency attenuates diabetic peripheral neuropathy in mice. Diabetes. 2019;68(11):2120‐2130. 10.2337/db18-1233 31439642 PMC6804630

[11] Henninger N , Bouley J , Sikoglu EM , et al. Attenuated traumatic axonal injury and improved functional outcome after traumatic brain injury in mice lacking Sarm1. Brain. 2016;139(Pt 4):1094‐1105. 10.1093/brain/aww001 26912636 PMC5006226

[12] Hughes RO , Bosanac T , Mao X , et al. Small molecule SARM1 inhibitors recapitulate the SARM1(‐/‐) phenotype and allow recovery of a metastable pool of axons fated to degenerate. Cell Rep. 2021;34(1):108588. 10.1016/j.celrep.2020.108588 33406435 PMC8179325

[13] Maynard ME , Redell JB , Zhao J , et al. Sarm1 loss reduces axonal damage and improves cognitive outcome after repetitive mild closed head injury. Exp Neurol. 2020;327:113207. 10.1016/j.expneurol.2020.113207 31962129 PMC7959192

[14] Choi HMC , Li Y , Suraj D , et al. Autophagy protein ULK1 interacts with and regulates SARM1 during axonal injury. Proc Natl Acad Sci USA. 2022;119(47):e2203824119. 10.1073/pnas.2203824119 36375051 PMC9704737

[15] Miao X , Wu Q , Du S , et al. SARM1 promotes neurodegeneration and memory impairment in mouse models of Alzheimer's disease. Aging Dis. 2024;15(1):390‐407. 10.14336/ad.2023.0516-1 37307837 PMC10796105

[16] Ding C , Wu Y , Dabas H , Hammarlund M . Activation of the CaMKII‐Sarm1‐ASK1–p38 MAP kinase pathway protects against axon degeneration caused by loss of mitochondria. eLife. 2022;11:e73557. 10.7554/eLife.73557 35285800 PMC8920508

[17] Ge YJ , Ou YN , Deng YT , et al. Prioritization of drug targets for neurodegenerative diseases by integrating genetic and proteomic data from brain and blood. Biol Psychiatry. 2023;93(9):770‐779. 10.1016/j.biopsych.2022.11.002 36759259 PMC12272336

[18] Hikosaka K , Yaku K , Okabe K , Nakagawa T . Implications of NAD metabolism in pathophysiology and therapeutics for neurodegenerative diseases. Nutr Neurosci. 2021;24(5):371‐383. 10.1080/1028415x.2019.1637504 31280708

[19] Jin L , Zhang J , Hua X , et al. Astrocytic SARM1 promotes neuroinflammation and axonal demyelination in experimental autoimmune encephalomyelitis through inhibiting GDNF signaling. Cell Death Dis. 2022;13(9):759. 10.1038/s41419-022-05202-z 36055989 PMC9440144

[20] Zhang J , Jin L , Hua X , et al. SARM1 promotes the neuroinflammation and demyelination through IGFBP2/NF‐κB pathway in experimental autoimmune encephalomyelitis mice. Acta Physiologica. 2023;238(2):e13974. 10.1111/apha.13974 37186158

[21] Bloom AJ , Mao X , Strickland A , Sasaki Y , Milbrandt J , DiAntonio A . Constitutively active SARM1 variants that induce neuropathy are enriched in ALS patients. Mol Neurodegener. 2022;17(1):1. 10.1186/s13024-021-00511-x 34991663 PMC8739729

[22] Bratkowski M , Burdett TC , Danao J , et al. Uncompetitive, adduct‐forming SARM1 inhibitors are neuroprotective in preclinical models of nerve injury and disease. Neuron. 2022;110(22):3711‐3726.e16. 10.1016/j.neuron.2022.08.017 36087583

[23] Shi Y , Kerry PS , Nanson JD , et al. Structural basis of SARM1 activation, substrate recognition, and inhibition by small molecules. Mol Cell. 2022;82(9):1643‐1659.e10. 10.1016/j.molcel.2022.03.007 35334231 PMC9188649

[24] Essuman K , Summers DW , Sasaki Y , et al. TIR domain proteins are an ancient family of NAD(+)‐consuming enzymes. Curr Biol. 2018;28(3):421‐430.e4. 10.1016/j.cub.2017.12.024 29395922 PMC5802418

[25] Brace EJ , Essuman K , Mao X , et al. Distinct developmental and degenerative functions of SARM1 require NAD^+^ hydrolase activity. PLoS Genet. 2022;18(6):e1010246. 10.1371/journal.pgen.1010246 35737728 PMC9223315

[26] Gürtler C , Carty M , Kearney J , et al. SARM regulates CCL5 production in macrophages by promoting the recruitment of transcription factors and RNA polymerase II to the Ccl5 promoter. J Immunol. 2014;192(10):4821‐4832. 10.4049/jimmunol.1302980 24711619 PMC4021400

[27] Jiang Y , Liu T , Lee CH , Chang Q , Yang J , Zhang Z . The NAD(+)‐mediated self‐inhibition mechanism of pro‐neurodegenerative SARM1. Nature. 2020;588(7839):658‐663. 10.1038/s41586-020-2862-z 33053563

[28] Xu K , Zhong G , Zhuang X . Actin, spectrin, and associated proteins form a periodic cytoskeletal structure in axons. Science. 2013;339(6118):452‐456. 10.1126/science.1232251 23239625 PMC3815867

[29] Guss EJ , Akbergenova Y , Cunningham KL , Littleton JT . Loss of the extracellular matrix protein perlecan disrupts axonal and synaptic stability during Drosophila development. eLife. 2023;12:e88273. 10.7554/eLife.88273 PMC10328508 37368474

[30] Kong L , Valdivia DO , Simon CM , et al. Impaired prenatal motor axon development necessitates early therapeutic intervention in severe SMA. Sci Transl Med. 2021;13(578):eabb6871. 10.1126/scitranslmed.abb6871 33504650 PMC8208236

[31] Simkins TJ , Duncan GJ , Bourdette D . Chronic demyelination and axonal degeneration in multiple sclerosis: pathogenesis and therapeutic implications. Curr Neurol Neurosci Rep. 2021;21(6):26. 10.1007/s11910-021-01110-5 33835275

[32] Cervellini I , Galino J , Zhu N , Allen S , Birchmeier C , Bennett DL . Sustained MAPK/ERK activation in adult Schwann cells impairs nerve repair. J Neurosci. 2018;38(3):679‐690. 10.1523/jneurosci.2255-17.2017 29217688 PMC5777114

[33] Mietto BS , Jhelum P , Schulz K , David S . Schwann cells provide iron to axonal mitochondria and its role in nerve regeneration. J Neurosci. 2021;41(34):7300‐7313. 10.1523/jneurosci.0900-21.2021 34272312 PMC8387113

[34] Alexandris AS , Lee Y , Lehar M , et al. Traumatic axonopathy in spinal tracts after impact acceleration head injury: ultrastructural observations and evidence of SARM1‐dependent axonal degeneration. Exp Neurol. 2023;359:114252. 10.1016/j.expneurol.2022.114252 36244414 PMC10321775

[35] Hsu JM , Kang Y , Corty MM , Mathieson D , Peters OM , Freeman MR . Injury‐induced inhibition of bystander neurons requires dSarm and signaling from glia. Neuron. 2021;109(3):473‐487.e5. 10.1016/j.neuron.2020.11.012 33296670 PMC7864878

[36] Li Y , Pazyra‐Murphy MF , Avizonis D , et al. Sarm1 activation produces cADPR to increase intra‐axonal Ca++ and promote axon degeneration in PIPN. J Cell Biol. 2022;221(2):e202106080. 10.1083/jcb.202106080 34935867 PMC8704956

[37] Sasaki Y , Nakagawa T , Mao X , DiAntonio A , Milbrandt J . NMNAT1 inhibits axon degeneration via blockade of SARM1‐mediated NAD(+) depletion. eLife. 2016;5:e19749. 10.7554/eLife.19749 27735788 PMC5063586

[38] Yaku K , Okabe K , Nakagawa T . NAD metabolism: implications in aging and longevity. Ageing Res Rev. 2018;47:1‐17. 10.1016/j.arr.2018.05.006 29883761

[39] Osterloh JM , Yang J , Rooney TM , et al. dSarm/Sarm1 is required for activation of an injury‐induced axon death pathway. Science. 2012;337(6093):481‐484. 10.1126/science.1223899 22678360 PMC5225956

[40] DiAntonio A , Milbrandt J , Figley MD . The SARM1 TIR NADase: mechanistic similarities to bacterial phage defense and toxin‐antitoxin systems. Front Immunol. 2021;12:752898. 10.3389/fimmu.2021.752898 34630431 PMC8494770

[41] Wang Q , Zhang S , Liu T , et al. Sarm1/Myd88‐5 regulates neuronal intrinsic immune response to traumatic axonal injuries. Cell Rep. 2018;23(3):716‐724. 10.1016/j.celrep.2018.03.071 29669278

[42] Carty M , Bowie AG . SARM: from immune regulator to cell executioner. Biochem Pharmacol. 2019;161:52‐62. 10.1016/j.bcp.2019.01.005 30633870

[43] Gerdts J , Brace EJ , Sasaki Y , DiAntonio A , Milbrandt J . SARM1 activation triggers axon degeneration locally via NAD⁺ destruction. Science. 2015;348(6233):453‐457. 10.1126/science.1258366 25908823 PMC4513950

[44] Marion CM , McDaniel DP , Armstrong RC . Sarm1 deletion reduces axon damage, demyelination, and white matter atrophy after experimental traumatic brain injury. Exp Neurol. 2019;321:113040. 10.1016/j.expneurol.2019.113040 31445042

[45] Zhu C , Li B , Frontzek K , Liu Y , Aguzzi A . SARM1 deficiency up‐regulates XAF1, promotes neuronal apoptosis, and accelerates prion disease. J Exp Med. 2019;216(4):743‐756. 10.1084/jem.20171885 30842236 PMC6446871

[46] Liu H , Zhang J , Xu X , et al. SARM1 promotes neuroinflammation and inhibits neural regeneration after spinal cord injury through NF‐κB signaling. Theranostics. 2021;11(9):4187‐4206. 10.7150/thno.49054 33754056 PMC7977471

[47] Lin CW , Hsueh YP . Sarm1, a neuronal inflammatory regulator, controls social interaction, associative memory and cognitive flexibility in mice. Brain Behav Immun. 2014;37:142‐151. 10.1016/j.bbi.2013.12.002 24321214

[48] Lehmann S , Loh SH , Martins LM . Enhancing NAD(+) salvage metabolism is neuroprotective in a PINK1 model of Parkinson's disease. Biol Open. 2017;6(2):141‐147. 10.1242/bio.022186 28011627 PMC5312101

[49] Chen CY , Lin CW , Chang CY , Jiang ST , Hsueh YP . Sarm1, a negative regulator of innate immunity, interacts with syndecan‐2 and regulates neuronal morphology. J Cell Biol. 2011;193(4):769‐784. 10.1083/jcb.201008050 21555464 PMC3166868

[50] Sorrentino V , Romani M , Mouchiroud L , et al. Enhancing mitochondrial proteostasis reduces amyloid‐β proteotoxicity. Nature. 2017;552(7684):187‐193. 10.1038/nature25143 29211722 PMC5730497

[51] Ghosh D , Levault KR , Brewer GJ . Relative importance of redox buffers GSH and NAD(P)H in age‐related neurodegeneration and Alzheimer disease‐like mouse neurons. Aging cell. 2014;13(4):631‐640. 10.1111/acel.12216 24655393 PMC4116450

[52] Du H , Guo L , Yan S , Sosunov AA , McKhann GM , Yan SS Early deficits in synaptic mitochondria in an Alzheimer's disease mouse model. Proc Natl Acad Sci USA. 2010;107(43):18670‐18675. 10.1073/pnas.1006586107 20937894 PMC2972922

[53] Li WH , Huang K , Cai Y , et al. Permeant fluorescent probes visualize the activation of SARM1 and uncover an anti‐neurodegenerative drug candidate. eLife. 2021;10:e67381. 10.7554/eLife.67381 33944777 PMC8143800

[54] Lu Q , Zhang Y , Botchway BOA , Huang M , Liu X . Syntaphilin inactivation can enhance axonal mitochondrial transport to improve spinal cord injury. Mol Neurobiol. 2023;60(11):6556‐6565. 10.1007/s12035-023-03494-6 37458986

[55] Marzan DE , Brügger‐Verdon V , West BL , Liddelow S , Samanta J , Salzer JL . Activated microglia drive demyelination via CSF1R signaling. GLIA. 2021;69(6):1583‐1604. 10.1002/glia.23980 33620118 PMC9250806

[56] Sarkar A , Dutta S , Sur M , Chakraborty S , Dey P , Mukherjee P . Early loss of endogenous NAD(+) following rotenone treatment leads to mitochondrial dysfunction and Sarm1 induction that is ameliorated by PARP inhibition. FEBS J. 2023;290(6):1596‐1624. 10.1111/febs.16652 36239430

[57] Calabrese V , Renis M , Calderone A , Russo A , Barcellona ML , Rizza V . Stress proteins and SH‐groups in oxidant‐induced cell damage after acute ethanol administration in rat. Free Radic Biol Med. 1996;20(3):391‐397. 10.1016/0891-5849(95)02095-0 8720910

[58] Mancuso C , Capone C , Ranieri SC , et al. Bilirubin as an endogenous modulator of neurotrophin redox signaling. J Neurosci Res. 2008;86(10):2235‐2249. 10.1002/jnr.21665 18338802

[59] Calabrese V , Cornelius C , Dinkova‐Kostova AT , Calabrese EJ , Mattson MP . Cellular stress responses, the hormesis paradigm, and vitagenes: novel targets for therapeutic intervention in neurodegenerative disorders. Antioxid Redox Signaling. 2010;13(11):1763‐1811. 10.1089/ars.2009.3074 PMC2966482 20446769

